# Psychological distress in hospitalized patients without COVID-19 symptoms: the role of fear of infection and remote contact with informal caregivers

**DOI:** 10.1017/S0033291720005012

**Published:** 2020-12-04

**Authors:** Valentina Colonnello, Gloria Leonardi, Marina Farinelli, Erik Bertoletti, Paolo M. Russo

**Affiliations:** 1 Department of Experimental, Diagnostic and Specialty Medicine, University of Bologna, 40127, Bologna, Italy; 2 Santa Viola Hospital, Colibrì Consortium, 40133, Bologna, Italy; 3 Clinical Psychology Service, Villa Bellombra Rehabilitation Hospital, Colibrì Consortium, 40136, Bologna, Italy

Dear Editor:

Recent studies have highlighted the psychological distress associated with COVID-19 in specific populations, from patients hospitalized with suspected COVID-19 infection (Wesemann et al., 2020) to clinically stable patients with COVID-19 (Bo et al., 2020). Surprisingly, however, the psychological distress among patients hospitalized for illnesses unrelated to COVID-19 has gone largely – and disproportionately – uninvestigated.

Italian hospitals have continued to admit patients for pathologies unrelated to COVID-19. For these patients, hospitalization occurs under unusual and unexpected conditions: a strict adherence to safety measures against COVID-19 and a substantial or complete disruption of direct social contact with informal caregivers. In fact, national pandemic safety guidelines require that in regions with higher numbers of COVID cases, visitors have limited access to hospitals (Italian Ministry of Health, 2020), which means patients cannot receive direct social support from their loved ones. Texts, telephone and video calls are the main ways patients can communicate with their caregivers. Thus, the profound innate need to receive comfort from emotionally significant others when stressed and feeling unsafe (Panksepp & Biven, 2012) remains unmet. Although patients without COVID-19 receive information about a hospital's COVID-19 prevention measures, they may still experience psychological distress related to a fear of COVID-19 because they know the hospital may have COVID-19 patients.

This raises questions about the relationship between fear of being infected by COVID-19 and psychological distress in patients without COVID-19. We hypothesized that in patients without COVID-19, fear of COVID-19 is associated with enhanced psychological distress regarding separation from caregivers (i.e. safety figures), negative expectations about their treatment, and poorer quality of wake–sleep rhythm regulation.

Positive social interactions enhance one's sense of safety and comfort and act as a protective factor for health (Colonnello, Petrocchi, Farinelli, & Ottaviani, 2017). Additionally, maintaining remote social contacts through mobile devices has been advocated as a way to counteract psychological distress (Fino, Fino, Mazzetti, & Russo, 2020). Thus, we also hypothesized that patients having more social contact, even if through remote means, experience less psychological distress.

To test these hypotheses, in March–July 2020, we recruited all non-COVID-19 patients admitted to two hospitals in Emilia Romagna, an Italian region hard hit by COVID-19 outbreak. Patients in these hospitals were not allowed to receive visitors. On day 4 or 5 after hospital admission, all participants signed a consent form and completed the Mini Sleep Questionnaire measuring the quality of wakefulness and sleep (MSQ; Natale, Fabbri, Tonetti, & Martoni, 2014), the Stanford Expectation Treatment Scale (SETS; Younger, Gandhi, Hubbard, & Mackey, 2012) measuring positive and negative expectations about one's treatment, and an ad-hoc developed 5-point Likert set of questions about COVID-19 measuring (1) the fear of contracting COVID-19 infection in the hospital, measured by two interrelated items (e.g. ‘I am worried about becoming infected with COVID-19’); (2) separation distress, measured by three items (e.g. ‘Not being allowed to stay with my caregiver makes me sad’); and (3) the number of contacts (i.e. calls, video calls, and text conversations) with their caregiver during hospitalization. In addition, participants completed the Hospital Anxiety and Depression Scale (HADS; Costantini et al., 1999) to control for the possible role of hospitalization-related anxiety and depression in patients' distress. The Emilia Romagna Ethical Committee approved these procedures (IRB numbers: 264-2020-OSS-AUSLBO and 265-2020-OSS-AUSLBO).

We conducted a series of multiple regression analyses on the data from 100 patients (age: 79 ± 11.29 years; 70 women) with various clinical conditions, mainly fractures (32%), heart disease (9%), stroke (6%), and pneumonia (6%). Dependent variables were distress levels due to separation distress, wakefulness and sleep quality, and treatment expectations. Independent variables were Fear of COVID-19 and Number of contacts with caregivers, controlling for sex, age, and HADS scores.

Patients experiencing more depression (*β* = 0.31, *p* = 0.003) and fear of COVID-19 (*β* = 0.49, *p* < 0.0001) reported more separation distress (*R*^2^_adj_ = 0.34, *F*_(6,93)_ = 9.55, *p* < 0.0001). In addition, anxiety (*β* = 0.44, *p* = 0.001), fear of COVID-19 (*β* = 0.27, *p* = 0.004) and, inversely, social contacts (*β* = −0.19, *p* = 0.046) predicted wakefulness problems (*R*^2^_adj_ = 0.21, *F*_(6,93)_ = 5.46, *p* < 0.0001). Sleep problems (*R*^2^_adj_ = 0.14, *F*_(6,93)_ = 3.69, *p* < 0.002) were predicted by anxiety (*β* = 0.28, *p* = 0.018) and fear of COVID-19 (*β* = 0.32, *p* = 0.001).

Regarding treatment expectations, remote social contact with the main caregiver was the only factor that predicted positive expectations about one's treatment (*R*^2^_adj_ = 0.20, *F*(6,93) = 5.20, *p* = 0.0001; contact: *β* = 0.38, *p* < 0.001). Negative expectations were predicted by anxiety and inversely by remote contacts (*R*^2^_adj_ = 0.12, *F*(6,93) = 3.25, *p* = 0.006; anxiety: *β* = 0.31, *p* = 0.010; contact: *β* = −0.23, *p* = 0.024).

Our results show that fear of COVID-19 and technology-mediated social interactions, along with anxiety and depression, predict central but distinct aspects of psychological distress in hospitalized patients without COVID-19. Remote social contact does not counteract patients' separation distress and sleep dysregulation, which are instead predicted by fearful alertness to the risk of being infected.

However, remote contact is associated with patients' quality of wakefulness and positive attitudes toward their clinical treatment, highlighting the role of social interactions as a protective factor for health. A parsimonious, though speculative, explanation of our findings is that communications between caregivers and patients might have been prevalently focused on supporting patients' positive attitudes toward their treatment.

Our results encourage a broadening of research attention to the psychological distress of hospitalized patients indirectly exposed to COVID-19. They also call for hospitals to invest in implementing psychology units aimed at promoting patients' positive attitudes toward their treatment by fostering and facilitating positive remote social contact with loved ones. Currently, hospitals that actually implement psychological support within a therapeutic strategy led by a clinical psychology service are rare in Italy. Given these findings, we advocate that addressing the needs of hospitalized patients without COVID-19 may lead to the optimization of healthcare during this pandemic, leaving no one to face hospitalization alone.
